# Short-Term Annoyance Due to Night-Time Road, Railway, and Air Traffic Noise: Role of the Noise Source, the Acoustical Metric, and Non-Acoustical Factors

**DOI:** 10.3390/ijerph18094647

**Published:** 2021-04-27

**Authors:** Sarah Weidenfeld, Sandra Sanok, Rolf Fimmers, Marie-Therese Puth, Daniel Aeschbach, Eva-Maria Elmenhorst

**Affiliations:** 1 German Aerospace Center (DLR), Institute of Aerospace Medicine, 51170 Cologne, Germany; sandra.sanok@dlr.de (S.S.); daniel.aeschbach@dlr.de (D.A.); eva-maria.elmenhorst@dlr.de (E.-M.E.); 2 Institute for Occupational and Social Medicine, Medical Faculty, RWTH Aachen University, 52074 Aachen, Germany; 3 Department of Medical Biometry, Informatics and Epidemiology (IMBIE), Faculty of Medicine, University of Bonn, 53127 Bonn, Germany; rolf.fimmers@ukbonn.de (R.F.); puth@imbie.uni-bonn.de (M.-T.P.); 4 Division of Sleep and Circadian Disorders, Brigham and Women’s Hospital, Boston, MA 02115, USA; 5 Division of Sleep Medicine, Harvard Medical School, Boston, MA 02115, USA

**Keywords:** road traffic noise, railway noise, aircraft noise, annoyance, *L_Aeq_*, number of events, field study, exposure–response curve

## Abstract

Field studies on traffic noise-induced annoyance have predominantly used estimated outside noise levels. We intended to complement existing knowledge with exposure–response relationships that are based on precise indoor noise measurements. Acoustic recordings inside the bedrooms of nightly road traffic and annoyance ratings in the following morning were obtained from 40 suburban residents (mean age 29.1 years ± 11.7; 26 females). We derived exposure–response functions for the probability to be “annoyed at least a little” (*%LA*). Further analyses compared data from the current study with those from two earlier studies on railway and aircraft noise. Annoyance increased with the number of traffic events and the equivalent sound pressure level. The inclusion of non-acoustical factors (such as assessment of road transport) improved the prediction considerably. When comparing the different traffic noise sources, *%LA* was higher for road than for air traffic at a given *L_Aeq,night_*, but higher for road and railway than for air traffic at a given number of noise events. Acoustical as well as non-acoustical factors impact short-term annoyance induced by road, railway, and air traffic. Annoyance varies across noise sources, which may be due to differences in acoustical characteristics or in the temporal noise distribution throughout the night.

## 1. Introduction

Environmental noise is a widespread and intrusive phenomenon of everyday life. It is often accompanied by perceived displeasure, irritation, and discomfort [1], and is associated with health risk, sleep disturbance, and annoyance [2]. In particular, annoyance over a longer time is regarded as an effect modifier of the relationship between noise and health risks [3]. Annoyance reactions are caused by repeated disturbances by noise combined with a person’s emotional and cognitive response to the sound [4]. The assessment depends on the physical characteristics of a sound, e.g., intensity, frequency, and duration. Additionally, it is influenced by the individual’s attitudes, judgements, personality traits, and the context in which it occurs [5]. These non-acoustical variables are the reason why the same sound might be perceived by one person as a pleasure and by another person as noise. Numerous earlier studies affirm and highlight, in addition to the noise event itself and its acoustical characteristics, the effect of non-acoustical variables on long-term annoyance [5,6,7].

With a growing transport network and increasing mobility of modern societies, it is not surprising that noise from road traffic, aircraft, and railway causes a large proportion of annoyance in the population. To describe the relationship between traffic noise and long-term annoyance, exposure–response curves as developed by Miedema and Oudshoorn [8] are widely accepted and are used for noise assessment by the European Commission [9]. The majority of studies focusing on the relationship between traffic noise and long-term annoyance are based on large-scale field surveys assessing the effect of noise exposure across a long period, for instance, the past 12 months (e.g., References [10,11]). On the one hand, large-scale field studies on community long-term annoyance enhance the validity and representative nature of the outcome, while on the other hand they lack precise acoustical data collection. The long-term annoyance responses are usually related to estimated noise levels of noise maps or measurements outside at the house façade. The lack of precise indoor measurements is a well-known shortcoming of these studies. Laboratory studies offer the opportunity to combine precise acoustical settings with short-term annoyance assessment, but it has been shown that the effect of noise is often overestimated in these settings [12]. Here, we therefore aimed at applying precise acoustical measurement techniques in an ecologically valid field setting.

Road traffic is the main source of environmental noise in cities and the overall vehicle use has increased by approximately 32% from 1991 to 2017 in Germany [13]. As a result, noise-free periods in urban areas are rare during the day and tend to diminish during night-time [14]. Since previous studies on road traffic rather focused on motorways with dense traffic, we intended to explore suburban areas with moderate traffic density at night. Previous studies have already proven that the A-weighted energy equivalent continuous sound pressure level, *L_Aeq_* in dB, which averages the sound energy of all traffic noise events over a specified time [14], is an important acoustical predictor of traffic noise-induced annoyance [15,16,17]. It is a preferred parameter to describe fluctuating sound levels. This parameter considers frequency, duration, and intensity of all noise events [18]. Recent studies have already shown that besides the *L_Aeq_*, the number of noise events is an important acoustical predictor for short-term annoyance due to railway and aircraft noise [16,19]. Therefore, we hypothesized that apart from the *L_Aeq_* for areas with moderate traffic density—due to its intermittent nature—the number of pass-bys may also be an important predictor for road traffic noise-induced short-term annoyance. Likewise, we expected non-acoustical variables to influence annoyance.

Most of the studies mentioned above refer to long-term annoyance, which describes a general feeling that has evolved over a longer period, at least several weeks. As a primary effect, noise during the night can disrupt sleep, which may lead to noise complaints on the following day as a secondary reaction [20]. Porter et al. [21] declare those next-day effects as short-term annoyance that may be an accumulation of negative feelings due to a disturbed night. Throughout this paper, we refer to annoyance as a short-term reaction which describes the feeling after a night with noise exposure. According to Guski et al. [1] and Bartels et al. [22], it is unclear whether and how respondents integrate their short-term experience over time into a long-term retrospective statement. Porter et al. [21] have elucidated the difference between short-term annoyance as a next-day effect and long-term annoyance as a chronic effect of noise over weeks, months, or years. However, despite the different time dimensions, they claimed that both share the same causes and characteristics. Since the number of studies on short-term annoyance is very small and there is a clear research gap of field studies on annoyance induced by road traffic noise, we intended to compare our findings on short-term annoyance with those on long-term annoyance.

The current study was conducted within the framework of a large program project (“Transport Development and Environment”) by the German Aerospace Center, the purpose of which was to develop an instrument to project the trends of passenger and commercial transport in Germany and of the worldwide air traffic until the year 2030. Consequences for the environment and people were also aspects of the project. Within a subproject on noise propagation and effects, one work package examined the impact of acoustical parameters on the objective sleep quality (data not shown here), and on short-term annoyance using precise measurements on site, i.e., in participants’ homes. Our intention was to derive ecologically valid exposure–response relationships between road traffic noise and short-term annoyance. Furthermore, we explored the effect of the traffic noise source on short-term annoyance. To this end, we pooled datasets from the current study and two previous studies on air and railway traffic noise [16,17] and carried out additional analyses. The similar approach of these three studies allowed for a ranking of annoyance probabilities depending on the noise source.

## 2. Materials and Methods

### 2.1. Residential Areas and Participants

At the beginning of the study, we pre-selected appropriate residential areas with moderate traffic density at night in the vicinity of Cologne and Bonn in Germany. A moderate traffic density was defined such that noises were attributable to separate events during the night. We chose roads taking the following criteria into account: (1) we selected roads in suburban areas with a speed limit of 30 or 50 km/h, (2) we excluded roads with conditions that might interrupt the pass-bys of road vehicles or encourage the development of vehicle columns (e.g., traffic lights or roundabouts), (3) we chose roads with a surface made of conventional asphalt concrete in generally good condition, and (4) we ensured that road traffic was the dominant noise source (generally higher noise levels and higher number of events compared to other noise sources such as railway, industry, aircraft, etc.) to avoid acoustical interferences of the road traffic recordings and a high noise exposure due to multiple noise sources. We used Google maps and Google street view for initial selection and inspected it by local explorations and by acoustic measurements at residents’ houses. The residents gave a signed informed consent for the acoustic recording at their home. Road traffic load and its composition are provided as Supplementary Data (Table S1). Following this selection process, we called for participation through advertisement on placards that were posted in the respective streets and on approximately 11,000 leaflets that were distributed in the residents’ mailboxes. Additionally, information about the study was given online (Facebook, DLR homepage) for initial applications followed by a check via Google maps or on-site whether the respective roads comply with the inclusion criteria described above. After the residents applied for participation, they received a questionnaire that screened for good health, living conditions, and absence of sleep disturbances. We excluded all persons with criteria that might influence the objective sleep physiology (e.g., small children in the household). As a second step, we recorded the road traffic noise in the candidates’ bedroom in a test-night to identify all possible interfering noise (e.g., the sound of an air conditioner, loud snoring of the candidate or their partner). If the noise would impair the evaluability of the acoustical data during the study and could not be prevented, the candidates were excluded. To ensure that they possessed a normal hearing ability according to their age, we performed an audiometer screening (AD226b, Interacoustics). Here, too, we obtained a signed informed consent for the test-night.

From a total of 534 candidates, 42 individuals were appropriate to participate after the multi-stage selection process. The data of two persons were excluded due to the presence of periodic leg movements during sleep (PLMS). All participants had to live at least 12 months at their home address (M = 7.9 years, SD = 6.3) to avoid that an insufficient adaptation might influence the sleep or the annoyance response. Forty healthy adult individuals with normal hearing ability according to their age were enrolled to participate, including 26 women and 14 men (mean age = 29.1 years; SD = 11.7). Their age ranged from 18 to 61 years. The study was approved by the Ethics Committee of the Medical Association North Rhine. Prior to the study, all participants gave a signed informed consent according to the Declaration of Helsinki [23]. They received remuneration for every day of participation in the study.

### 2.2. Measurements of Noise Exposure

Data collection took place during summer/autumn of 2015 and 2016. Since the number of pass-bys differ with respect to the weekday, measurements were carried out at participants’ homes on five consecutive nights, including the weekend. The measurements included polysomnographic recordings at night (data not shown here) and assessments of annoyance in the morning. The first night served as adaptation to the unfamiliar setting. Participants had to keep a time in bed of 7 to 8 h duration. They were free to choose a bedtime between 22:00 and 23:30 h and a rising time between 6:30 and 8:00 h fixed throughout the examination nights. They were also free to choose the position of the bedroom window (open, tilted, closed) they preferred but were requested to not change it during the night. Road traffic noise exposure was measured in 45.5% of the study nights with closed windows, in 46.8% with tilted windows, and in 7.7% with open windows (detailed information is given in Supplementary Table S1).

The inside measurement of nocturnal road traffic noise was undertaken with Class-1 sound level meters (XL2 from NTi Audio). A microphone was placed inside the participants’ bedroom close to the bed’s headboard. The sound pressure level was recorded with A-weighting and time weighting fast-response in a one second interval. The acoustical recordings started automatically 15 min before their fixed bedtime and stopped 15 min after their rising time in the morning. In addition, a traffic counter (viacount II, via traffic controlling) was mounted at the street lamppost closest to the respective bedroom window. The radar detector technology provided information about the number of road traffic events, velocity, length, height, vehicle class (i.e., truck, transporter, motorbike, and car), and time. All participants lived in houses that directly faced a street (maximum distance to road approximately 22 m).

We used the information of the traffic counter to pre-classify the recorded road traffic noise events by a specially developed acoustic program. The program marks start as well as end of a road traffic noise event and determines the vehicle class. To ensure a valid and reliable scoring, the data were double-checked by human scorers. The scorers listened to the acoustical recordings via headphones, checking whether the automatically pre-classification and markings were set correctly. If necessary, they revised or added the markings manually. To limit the analysis of the recording to the participants’ time in bed, their exact bedtime and rising time were also marked manually. In addition to the *L_Aeq,night_*, we calculated 13 further acoustical variables per participant and night. The number of nocturnal road traffic noise events were derived from the traffic counter data.

### 2.3. Measurements of Subjective Responses

On each of the five consecutive mornings immediately after getting up, the participants completed a paper and pencil questionnaire which included a question on annoyance referring to the previous night: “Thinking about the last night, when you are at home, how much did noise from road traffic bother, disturb, or annoy you?” We used a five-point standardized equidistant response scale of Likert type (“1 = not at all”, “2 = slightly”, “3 = moderately”, “4 = very”, and “5 = extremely”). This scale was developed by the International Commission on Biological Effects of Noise (ICBEN) to provide internationally comparable measurements of annoyance [24]. To assess the subjectively perceived noise load due to nocturnal road traffic using the same response scale, the questionnaire also asked, “Thinking about the last night, when you are at home, how intensely did you hear the road traffic noise?” Furthermore, the participants estimated their subjective sleep quality with six different 100 mm long visual analogue scales [25] that referred to difficulties in falling asleep (easy–difficult), calmness of sleep (calm–restless), sleep duration (too short–too long, inversely coded for analysis), restoration (high–low), sleep depth (sound–shallow), and body movements (little–much). The values were added up and subtracted from 60 (the maximum overall score). Low values represent a decreased and high values an increased sleep quality.

To obtain further non-acoustical variables that might contribute to the development of annoyance responses, we used additional questionnaires at the beginning and at the end of the study. In this way, we gathered demographical data as well as psychological data that referred among others to the participants’ assessment of road transport (necessity, acceptability, eco-friendliness, economic importance: e.g., “Do you consider road transport in general as economically important?”) and their general adaptation to chronic road traffic noise exposure (“How well can you adapt to road traffic noise in general?”). For the psychological parameters that were measured by Likert scales with several items (range 3 to 13), we calculated Cronbach’s alpha. The internal consistency of the scale “assessment of road transport” improved appreciably after exclusion of the item “eco-friendliness”. The values of the remaining three five-point answering scales (from “1 = not at all” to “5 = extremely”) were added up so the final ‘assessment of road transport’ parameter ranged from 3 = negative to 15 = positive assessment. Cronbach’s alphas of all scales ranged between 0.75 and 0.83 and were classified as acceptable to good.

### 2.4. Statistical Analyses

We excluded all adaptation nights as well as four additional nights due to absence of annoyance data. Whereas the final dataset of the acoustical recordings consisted of 156 nights, the traffic counter data were reduced by 17 nights. As a result, 41,872 road traffic noise events that occurred throughout participants’ time in bed over 139 nights were taken into account.

We analyzed the data with the statistical program IBM SPSS Statistics version 21 (IBM Corp., Armonk, New York). The distribution of the annoyance ratings of all nights was left skewed. Only 1% (N = 2) of the participants felt extremely annoyed, 5% (N = 8) stated that they were very annoyed, 12% (N = 18) reported a moderate annoyance, and 31% (N = 48) chose the response option “slightly annoyed”, while 51% (N = 80) felt not at all annoyed. Due to the small proportion of moderately, very, and extremely annoyed persons, the widely used annoyance descriptors “highly annoyed” (*%HA*) and “annoyed” (*%A*) recommended by the European Commission [26] could not be applied. These descriptors define the percentage of persons with responses exceeding a value of 72 (for *%HA*) and 50 (for *%A*) on a 0 to 100 scale, respectively. Therefore, a binary variable was generated differentiating between subjects who were not at all annoyed (N = 80, value 0) and subjects who were slightly to extremely annoyed (N = 76, value 1). This corresponded to a statistical approach established by Miedema and Oudshoorn [8], who used a general rule to translate scales with different numbers of response categories (e.g., a 5-point scale) into the 0 to 100 scale and suggested a cutoff at the value 28 to account for the percentage of people who were annoyed at least a little (*%LA*).

We used Generalized Estimating Equations (GEE) with logistic regression to model the annoyance data. The GEE is an extension of Generalized Linear Models (GLM) and was established by Zeger and Liang [27]. It describes associations as well as interactions between the variables, determines the strengths of the effects, controls effects of confounding variables, and gives predictive estimates of the response variable at certain values of the explanatory variable [28]. “The estimated coefficients for the independent variables represent the slope (i.e., rate of change) of a function of the dependent variable per unit of change in the independent variable.” [29] (p. 47). Another parameter that describes the strength of the association is the odds ratio (OR). This parameter reflects the odds that a response occurs due to a specific exposure compared to the odds that it occurs in the absence of the exposure. The OR takes the value 1 when there is no effect of the independent variable on the odds of the outcome variable. An odds ratio less than 1 is related to lower odds, and conversely, an odds ratio of more than 1 indicates higher odds of response [30]. Repeated measurements of a subject are usually correlated and can be handled as a cluster. The GEE provides the opportunity to deal with those data [31]. The correlation within a cluster should be taken into account to avoid biased standard error estimators [28].

Based on the assumption that the relationship between the acoustical and non-acoustical parameters and the binary annoyance variable is nonlinear, we used logistic regression. Logistic regression estimates the probability for binary outcomes. Pan [32] introduced a valid criterion for GEE—the quasi-likelihood under independence model criterion (QIC) to choose the working correlation structure and to select the predictor variables. The smaller the value of this information criterion, the better the correlation structure and the model fit. Since the true correlation structure is unknown, we decided to choose the unstructured working correlation matrix according to the QIC.

In a first step, a univariable analysis was performed for the binary annoyance variable and each acoustical and non-acoustical variable. We chose every non-acoustical variable on the basis of a significant correlation with the binary annoyance variable (*p* < 0.05) for further multivariable analyses. This approach revealed the following non-acoustical candidates: (1) residential satisfaction, (2) general perception of loudness in the residential area, (3) subjective sleep quality, (4) general adaptation to chronic road traffic noise exposure, (5) assessment of road transport, (6) concerns about harmful effects of road transport, (7) subjectively perceived noise load in the previous night, (8) long-term annoyance due to road traffic noise in general, (9) long-term annoyance due to noise by passenger car transport, (10) long-term annoyance due to noise by heavy vehicles, (11) activity disturbances due to road traffic noise, and (12) sleep disturbances. In a second step, we calculated multivariable logistic regressions for the *L_Aeq,night_* and the number of nocturnal road traffic noise events, each in a separate model to analyze the impact of road traffic noise on short-term annoyance. We incrementally added the appropriate non-acoustical covariates in the multivariable models. We used the stepwise forward selection process and observed the QIC to select the appropriate predictors. Those covariates which had still a significant effect (*p* < 0.05) and decreased the QIC the most remained in the model. Only after any further variable missed significance and did not contribute to the model fit was the including completed. All possible two-way interactions between the selected variables and non-linear transformations were tested. None improved the model fit. As a final step, a multiple regression analysis was used to test for multicollinearity. The resulting variance inflation factors (VIF) were < 5, which precludes any critical intercorrelation among the independent variables in the model [33].

The DLR Institute of Aerospace Medicine had previously conducted two field studies to investigate the effect of nocturnal railway [16] and aircraft noise [17] on short-term annoyance of residents at their homes in the vicinity of Cologne and Bonn. The railway noise study [16] was undertaken from 2008 to 2009 with 33 participants (22 female) between 22 and 68 years of age (M = 36.2, SD = 10.3). Sixty-four residents (35 female) at the age of 19 to 61 (M = 37.4, SD = 12.8) participated in the aircraft noise study from 2001 to 2002 [17]. In both studies, the measurements were carried out on nine consecutive nights, including one adaptation night. We excluded the adaptation nights, as well as nights without acoustical recordings and questionnaire data. Although the psychological variables (i.e., traffic noise annoyance, subjective perception of noise load, general adaptation to chronic noise exposure of the traffic source, as well as perceived necessity, health hazard, and avoidability of the traffic source) of both studies were assessed by the response scale “not” to “very”, we regarded them as comparable with the response scale of the current study on road traffic (“not at all” to “extremely”) due to their semantic relationship. The acoustical recordings were also measured inside the bedroom with the appropriate time weighting depending on the noise source—railway noise events by the fast-response and aircraft noise by slow-response. The calculation of the *L_Aeq,nigh_t* in the air traffic study considered only those traffic noise events exceeding a maximum sound pressure level of 35 dB.

We combined the data of these two studies with those from the present study on road traffic (descriptive statistics are given in Table 1) and used the identical statistical approach as mentioned above to account for the probability to be annoyed at least a little (*%LA*). According to the QIC, we chose the exchangeable working correlation matrix for GEE analyses. To derive exposure–response curves for every traffic noise source, the models contain a variable which refers to the respective kind of traffic noise source. Road traffic noise and railway noise were compared with aircraft noise, which served as a reference. Out of 11 congruent non-acoustical variables between the 3 studies, the following were significantly correlated (*p* < 0.05) with the binary annoyance variable and were considered in the stepwise forward selection process: subjective perception of noise load, general adaptation to chronic noise exposure of the traffic source, and health hazard.

## 3. Results

### 3.1. Descriptive Results

**Figure 1:**
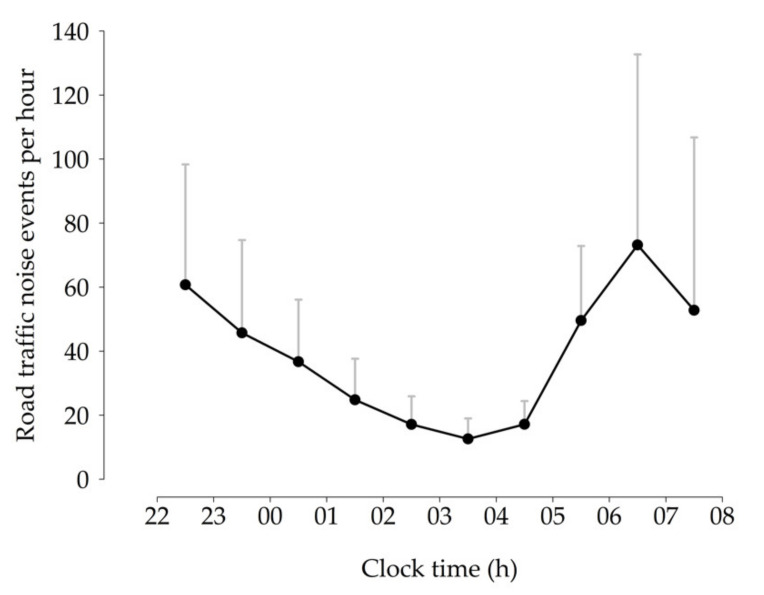
Hourly distribution of road traffic noise events during participants’ time in bed based on data provided by a traffic counter. Vertical lines correspond to standard deviations. The number of events per night were averaged within and across participants (N = 40).

Participants were exposed to an average of 297 passing vehicles per night, with a *L_Aeq,night_* of approximately 28 dB, as measured inside the bedroom (Table 1). Figure 1 displays the hourly distribution of road traffic noise events during participants’ time in bed. The number of recorded pass-bys within the morning hour (6:00–7:00 h) was reduced since participants were free to choose an individual time in bed (between 22:00 and 8:00 h and with a duration of 7 to 8 h), such that not all participants were still in bed after 7:00 h.

### 3.2. Prediction of Short-Term Annoyance by Road Traffic Noise

The univariable analyses showed a significant effect of the *L_Aeq,night_* (*p* = 0.037, OR = 1.058, 95% CI (1.003, 1.116)), whereas all other acoustical metrics had no effect on the binary annoyance variable alone. According to the QIC, the best model fit was yielded by the number of nocturnal road traffic noise events (QIC = 192.414). The long-term annoyance by road traffic noise referring to the past 12 months correlated positively with the binary short-term annoyance variable (*p* = 0.004, OR = 2.502, 95% CI (1.337, 4.682)). Results for all 15 acoustical variables as well as the 12 non-acoustical candidates and their associations with the binary annoyance variable are shown as Supplementary Data (Tables S2 and S3).

**Figure 2:**
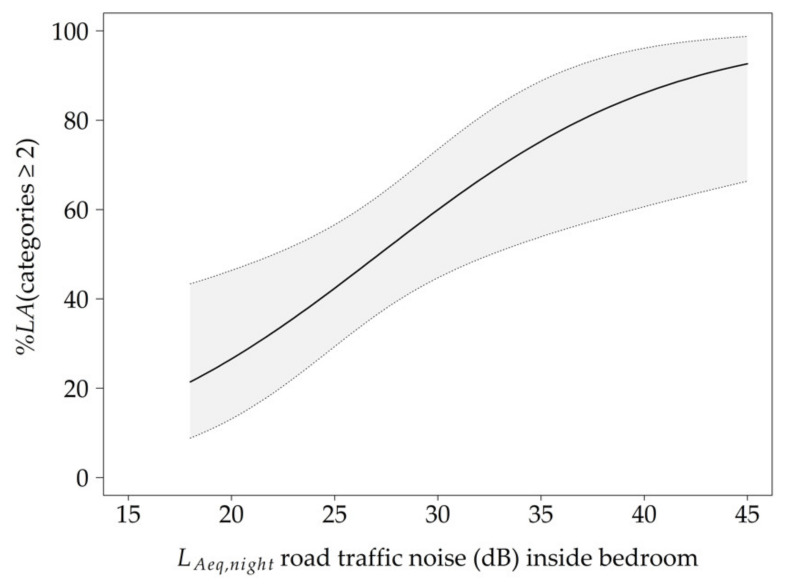
Exposure–response curve for the probability to be annoyed at least a little (*%LA*) due to the A-weighted energy equivalent sound level, *L_Aeq,night_,* of nocturnal road traffic noise measured inside the bedroom. Other covariates were set at their respective median (subjectively perceived noise load = 2; subjective sleep quality = 38.8). The grey area represents the 95% confidence interval.

The probability to be annoyed at least a little increased with rising *L_Aeq,night_* (see Table 2 for results of GEE 1 model). Additionally, *%LA* rose with an increase in subjectively perceived noise load during the previous night, whereas a high subjective sleep quality was associated with reduced annoyance. The average *L_Aeq,night_* inside the bedroom of approximately 28 dB led to 53 *%LA,* while the maximum recorded value of approximately 45 dB was associated with a 93% probability to be annoyed at least a little by road traffic (Figure 2).

**Figure 3:**
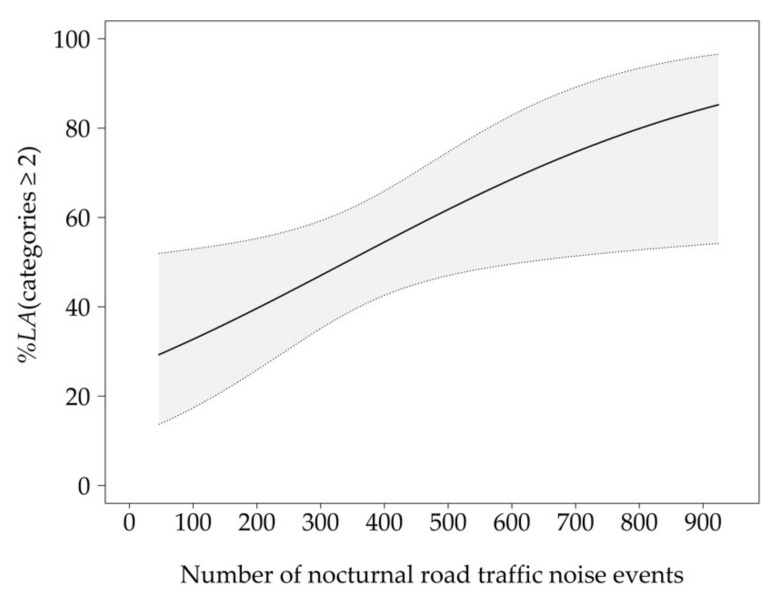
Exposure–response curve for the probability to be annoyed at least a little (*%LA*) due to the number of nocturnal road traffic noise events adjusted for the participants’ subjective assessment of road transport (median = 9). The grey area represents the 95% confidence interval.

As shown in GEE 2 (Table 2), an increase in the number of nocturnal noise events increased the probability to be annoyed at least a little (Figure 3). The model yielded the best data fit, when participants’ subjective assessment of road transport was added as a non-acoustical variable. A positive assessment of road transport decreased the probability for subjects to be annoyed at least a little. Approximately 297 pass-bys per night resulted in 47 *%LA*. The selection process did not include subjective sleep quality and subjectively perceived noise load for model 2, possibly due to the fact that they each correlated with the number of noise events (subjective sleep quality: r = −0.307, *p* < 0.001; perceived noise load: r = 0.252, *p* = 0.003). The lower QIC value of GEE 1 indicated a better model fit due to the inclusion of the non-acoustical factors compared to GEE 2.

### 3.3. Prediction of Short-Term Annoyance by Road, Railway, and Aircraft Noise

The pooled dataset included 137 participants and 76,611 noise events. Participants were exposed to an average of approximately 67 trains per night, with an indoor *L_Aeq,night_* of approximately 37 dB in the railway study (Table 1). In the aircraft noise study, an average of approximately 36 airplanes per night caused a *L_Aeq,night_* of approximately 25 dB measured inside the bedroom. We visualized the stepwise forward selection process in Table 3 (GEE 3, GEE 4, GEE 5) and Table 4 (GEE 6, GEE 7) to explore the effect of the specific noise sources on the binary annoyance variable. In a simple model (GEE 3) including only the three traffic noise sources, the probability to be annoyed at least a little was highest for railway noise. The latter did not differ from road traffic noise (*p* = 0.925, OR = 1.034, 95% CI (0.519, 2.056)), but differed from aircraft noise, which was least annoying.

**Figure 4:**
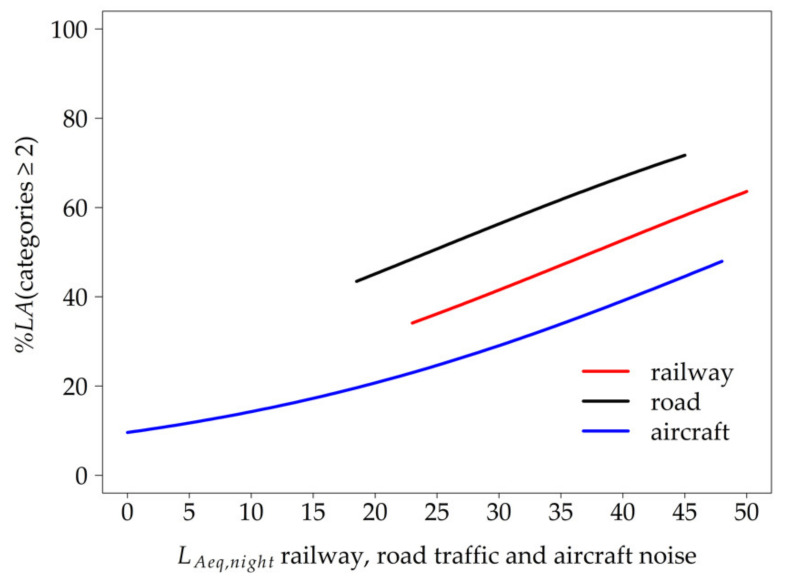
Probability to be annoyed at least a little (*%LA*) by nocturnal road traffic, railway, and aircraft noise expected by model GEE 5 as a function of the *L_Aeq,night_* measured inside the bedroom adjusted for general adaption to chronic noise exposure (median = 3).

In model GEE 4, the *L_Aeq,night_* measured indoors was added as a factor (Table 3). The higher the *L_Aeq,night_*, the higher the *%LA* for all traffic modes (non-significant interaction between *L_Aeq,night_* and traffic noise sources). When controlling for the general adaptation to noise, the model resulted in road traffic, but not railway traffic, being more annoying than aircraft traffic. (GEE 5, Figure 4).

**Figure 5:**
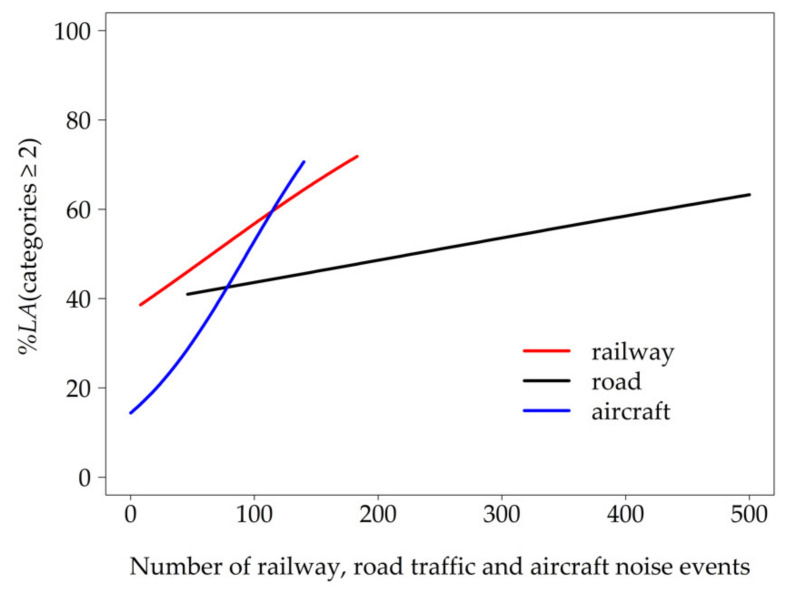
Probability to be annoyed at least a little (*%LA*) depending on the number of nocturnal road traffic, railway, and aircraft noise events expected by model GEE 7 adjusted for general adaptation to chronic noise exposure (median = 3).

The number of nocturnal noise events proved to be a significant acoustical predictor (GEE 6, Table 4) and its inclusion in the intermediate model explained the differences in *%LA* between the traffic modes. The best model fitting was achieved (GEE 7) when participants’ general adaptation to noise was included as a factor (lower *%LA* with increasing degree of adaptation, irrespective of the traffic mode). Since an interaction between general adaptation and traffic noise source was not significant and impaired the model fit, it was not included. When controlling for the number of nocturnal noise events, the probability to be annoyed at least a little was higher for railway than for air traffic and higher for road than for air traffic, while road traffic did not differ from railway (*p* = 0.924, OR = 1.074, 95% CI (0.248, 4.647)). The percentage of at least a little annoyed residents as predicted by the number of nocturnal railway, road traffic, and aircraft noise events (model GEE 7) is shown in Figure 5. There was an apparent interaction between road traffic and the number of nocturnal noise events. As long as the number of nocturnal traffic noise events was below 79, road traffic noise caused more annoyance reactions than air traffic. However, with an increasing number of nocturnal noise events, air traffic became more annoying than road traffic. The interaction between railway traffic and the number of events did not differ significantly from road traffic (*p* = 0.299, OR = 0.994, 95% CI (0.982, 1.006)) or air traffic.

## 4. Discussion

### 4.1. Effect of Road Traffic Noise on Short-Term Annoyance

For a better traceability, the main characteristics of our study and those used to back up the discussion and conclusions are summarized within the Supplementary Data (Table S4). The present study provides evidence that an increase in the *L_Aeq,night_* from road traffic measured inside the home environment results in a higher percentage of residents who are annoyed at least a little. This corresponds to previous findings on long-term annoyance based on estimated noise levels outside at the house façade (i.e., Reference [8]). In addition, our results showed that short-term annoyance probability (*%LA*) increased with the number of night-time vehicle pass-bys. Similarly, Jakovljevic et al. [15] have emphasized the number of noise events as an important characteristic of road traffic noise. They also highlighted the role of night-time road traffic noise exposure measured outside as a better predictor of high long-term annoyance levels in comparison to daytime exposure.

Measurements inside the participants’ bedroom enabled us to explore the effect of the noise levels reaching the ear. Locher et al. [34] emphasized the importance of the indoor sound levels in health studies. They criticized that the application of a constant correction factor for indoor levels “…is a very coarse estimate and does not take into account specific conditions of the dwelling situation, window opening behavior, and building characteristics” (p. 2). They pointed out that health effects can be biased in every direction by misclassification of the noise exposure. Bartels et al. [22] derived individualized noise metrics from the outside recordings that considered the participant’s whereabouts and window position to account for outdoor to indoor attenuation. They concluded that the individualized indoor metrics predict short-term annoyance ratings more precisely than the outdoor measurements. By measuring the actual indoor level in the present study, we were able to overcome these limitations and to minimize the probability of biased outcomes. Since the participants were free to choose the preferred window position (closed, tilted, open), the sound level inside may have been a consequence of their response to the traffic noise outside, which may have skewed the relationship between inside noise and annoyance. However, the window position did not significantly influence the binary annoyance variable in our study (data not shown). Based on the results by Müller [35], we assume that the outdoor noise level was attenuated by 13 dB(A) for open windows, 14 dB(A) for tilted windows, and 27 dB(A) for closed windows. Given the mean indoor *L_Aeq_* of approximately 28 dB and that the windows were mainly closed or tilted in our study, the mean outdoor level might have been between 42 and 55 dB(A). The German night-time immission limits (*L_Aeq_*) outside at the façade during construction or major changes to public roads are 49 dB(A) for pure residential areas and 54 dB(A) for residential areas mixed with commercial buildings [36].

Pennig et al. [16] and Quehl et al. [19] showed a significant effect of the number of railway and aircraft noise events on short-term annoyance and argued that the effect of traffic noise should not be evaluated exclusively on the basis of the *L_Aeq_*. Although the *L_Aeq_* considers the number of events, it does not give detailed information about the traffic composition and the temporal pattern. Since a few noisy events can result in the same *L_Aeq_* value as many less noisy events, it is possible that the different temporal patterns may result in different annoyance reactions. As many other authors, Gjestland and Gelderblom [37] emphasized that noise-induced annoyance is not unambiguously described by a cumulative metric such as the *L_Aeq_*. Their meta-analysis also showed a significant role of the number of aircraft movements derived from the survey reports and airport data in predicting long-term annoyance. At equal noise levels estimated for outside, annoyance increased with the number of events. The authors assumed that in comparison to many noise events at lower level, few but louder events might result in longer quiet inter-event intervals and thus lower annoyance [38]. Thus, the number of pass-bys as a separate acoustical predictor should also be taken into account for noise protection concepts and noise insulation. In our study, the unadjusted models that included only acoustical metrics and were calculated by univariable analysis revealed that the number of pass-bys yielded a better model fit than the *L_Aeq,night_* did. Suburban road traffic is typically composed of short isolated noise events during the night. In contrast, urban traffic is more dense, and is characterized by few noise-free intervals. This difference might explain why the number of events proved to be a more meaningful metric to predict annoyance in suburban residents.

The consideration of non-acoustical predictors is important for the explanation of variance in annoyance among the participants. They might explain why some individuals felt annoyed even when the exposure was low, whereas others were not annoyed even though the noise load was high [22]. The current study corroborates the importance of non-acoustical factors. The predictors in our study, selected by the best model fit, differed depending on the acoustical parameters (*L_Aeq,night_* vs. number of nocturnal events). Similar observations have been reported for aircraft noise-induced short-term annoyance [17].

The current study demonstrates an important association between sleep quality and short-term annoyance and supports the assumption of Porter et al. [21] that the accumulation of sleep disturbances leads to annoyance responses the next morning. Griefahn [39] has suggested that the subjective assessment of sleep quality is predominantly determined by the consciously perceived noise exposure and does not refer to physiological reactions while asleep. A study by Öhrström [40] which investigated the impact of road traffic noise measured outside on long-term annoyance reported that sleep disturbances during the time before sleep onset and the time before getting up are more annoying than during other hours. In our study, most pass-bys of road traffic occurred at the beginning (23:00 h to 24:00 h) and near the end of participants’ time in bed (5:00 h to 7:00 h). The morning hours exhibited a higher number of events without or with only short noise-free phases. The reduced sleep pressure in the morning hours might result in an increased awakening probability and therefore lead to a conscious perception of the high noise exposure. Our data are not suitable to test this hypothesis. This could be examined systematically in a laboratory setting. In accordance with Öhrström [41] and Pennig et al. [16], who found a correlation between the number of noise events and self-reported sleep disturbances, we revealed a significant relationship of the number of noise events with the subjective sleep quality.

When road transport was assessed as important, necessary, and acceptable, *%LA* decreased in our study. This effect has also been reported in the context of railway noise-induced long-term annoyance [42]. Importantly, this non-acoustical factor holds the potential to be improved by the respective authorities and transport operators. In the case of railway and air transport, authorities and institutions could foster transparency and involve residents in their decisions, which might change residents’ attitude towards the noise source and therefore the degree of annoyance. In contrast, given that many individuals who are exposed to road traffic noise are also car drivers—in particular in suburban areas as studied here—it may make it more difficult to change an individual’s attitude towards road transport.

The subjective perception of the noise load turned out to have a large impact on short-term annoyance (OR = 44.6) in the current study. This supports the assumption of Lam et al. [43] that an individual’s perception of loudness is a better predictor of long-term annoyance than an acoustical metric. Since the processing of sound depends on the subjective perception, this might explain why we and some other authors did not find significant effects on annoyance for the acoustical metrics alone, with exception of the *L_Aeq_* (i.e., Reference [16]). Nevertheless, it should be kept in mind that we measured a verbal expression of the residents’ perception. Schreckenberg and Schuemer [44] reported two non-acoustical variables that contributed more to the prediction of long-term annoyance than the *L_Aeq_*. Hence, they concluded that non-acoustical variables together with annoyance may reflect a general form of annoyance response to traffic noise.

### 4.2. Comparison of Road, Railway, and Aircraft Noise Effects on Short-Term Annoyance

Comparing the impact of the three major traffic noise sources—road, railway, and air traffic—on short-term annoyance, the probability to be annoyed at least a little varied not only depending on the respective noise source, but also depending on the acoustical metric. One might argue that the comparability to other traffic noise studies using “*%HA*” or “*%A*” might be limited. However, an analysis by Miedema and Oudshoorn [8], exploring different cut-off points for long-term annoyance scales (*%LA*, *%A*, and *%HA*), resulted in the same ranking of transportation modes irrespective of the cut-off point. Based on these findings, we assume that our results on *%LA* are comparable with those of studies on *%HA* or *%A*. While several studies have already proven that long-term annoyance responses differ by source [11,45], the latter has only been reported by a few studies. The ranking of traffic modes regarding their impact on short-term annoyance in laboratory studies changed when the maximum sound pressure level was taken into account instead of the *L_Aeq_* [46,47]. However, to our knowledge, the current study is the first to compare the effect of road traffic with that of railway and air traffic on short-term annoyance with respect to the number of noise events. Interestingly, when the number of events was low (< 79), *%LA* was higher for road traffic than for air traffic, whereas the reverse was observed with higher number of events. The result, that railway traffic did not differ significantly from air traffic when the effect of number of nocturnal events on short-term annoyance was taken into account, is in accordance with findings by Elmenhorst et al. [12].

The traffic modes showed a near-linear increase in *%LA* with increasing *L_Aeq,night_* in the order road traffic > air traffic. Annoyance due to railway traffic was not significantly different from air traffic. These findings on short-term annoyance differ from observations on long-term annoyance by Brink et al. [11], who ranked road traffic < railway traffic < aircraft noise. Our findings also differ from the European standard curves, which ranked railway < road traffic < aircraft noise [8]. However, the acoustical parameters of these studies refer to the exposure measured or calculated by propagation models outside at the house façade. Furthermore, unlike these studies whose exposure–response curves were based on separate models for each primary noise source, we derived the curves based on a pooled model, thus allowing for more direct comparisons. In a large German survey, road traffic was rated as the most annoying traffic noise source, followed by air and railway traffic [48]. However, again, comparisons with our study are difficult since the survey data related to a random sample of the general German population, irrespective of the respondents’ noise exposure.

Noise researchers argued that the different acoustical properties of noise sources such as temporal pattern, predictability, and frequency composition of noise events [49,50] might be responsible for differences in annoyance reactions. Versfeld and Vos [51] have demonstrated in a laboratory study that different spectral characteristics of vehicles caused by engine type and operating behavior affected short-term annoyance. While road traffic exhibited more variability (i.e., mopeds, cars, lorries), aircraft or railway noise varied less, and occurred according to a more regular time pattern [50]. In our study, the temporal pattern of air traffic at Cologne-Bonn differed from that of road traffic, with most over-flights occurring between 23:00 to 1:00 h and between 3:00 to 5:00 h [12]. In addition, air and railway traffic were characterized predominantly by single noise events and noise-free intervals in between, as opposed to the dense road traffic during the morning rush hour.

The higher participants rated their general adaptation to noise, irrespective of the noise source, the lower their annoyance level was. This finding is in line with studies on railway noise by Pennig et al. [16]. The authors noted that the adaptation to railway noise did not reflect an incremental process over time, but rather the individual ability to cope with traffic noise.

### 4.3. Limitations

The burden of extensive noise exposure measurements in the present study limited the number of participants that we were able to include. Moreover, the road traffic in the chosen suburban areas induced only low levels of short-term annoyance in affected residents. Nevertheless, we considered it relevant to address the question of noise effects in suburban areas (i) since a large proportion of the population lives in these areas and (ii) since research has predominantly focused on regions with high noise exposure.

We confirmed previous studies that found a direct relation between short-term and long-term annoyance [22,44]. However, since studies on long-term annoyance generally use an averaged exposure that is calculated for day and night with a 10 dB penalty (*L_dn_*) for the night-time, a direct comparison to our findings may be limited.

Even though data collection procedures were in large parts identical for the studies on road, railway, and aircraft noise, some differences in questionnaires limited the number of non-acoustical variables which were available for comparison. Slight differences also existed in calculating the *L_Aeq,night_*: in the present study on road and the earlier study on railway traffic, all events from the respective noise source contributed to the *L_Aeq,night_*, whereas in the study on aircraft noise, only events exceeding the threshold of 35 dB were taken into account. This might have resulted in slightly higher *L_Aeq,night_* values in the study on road traffic.

While established acoustical parameters like the *L_Aeq_* are quite stable in their correlation with annoyance, the impact of non-acoustical variables on annoyance can differ depending on the traffic noise source and on the examined population [52]. Therefore, our results should only cautiously be generalized to areas outside the Cologne and Bonn region.

## 5. Conclusions

The present study fills the gap for exposure–response relationships between nocturnal road traffic noise and short-term annoyance in suburban areas, taking the *L_Aeq,night_* and the number of nocturnal events assessed by precise measurements inside the residents’ home into account. Furthermore, it provides for the first time, to our knowledge, a direct comparison of the road, railway, and air traffic noise effects on short-term annoyance. The results support the assumption that both the *L_Aeq,night_* measured inside and the number of nocturnal events contribute to the development of annoyance reactions induced by different traffic noise sources. The data emphasize the significant role of non-acoustical variables for the prediction of short-term annoyance. Surprisingly, whether road, aircraft, or railway traffic was perceived as more annoying depended on the respective acoustical metric. Thus, caution is warranted when ranking traffic modes with respect to the degree of noise annoyance they cause. Since the examined sample is small and the moderate nocturnal road traffic of the investigated areas led to rather low annoyance levels, prospective investigations should be extended to a larger sample as well as include residential areas with dense road traffic.

## Figures and Tables

**Table 1:** Descriptive statistics of short-term annoyance and its acoustical and non-acoustical predictors.

	Traffic Noise Source								
	Road			Railway			Aircraft		
	Mean (SD)	Min.	Max.	Mean (SD)	Min.	Max.	Mean (SD)	Min.	Max.
*L_Aeq,night_* of traffic noise events indoors (dB)	27.86 (5.18)	18.5	44.7	36.62 (6.05)	23.1	49.3	24.88 (8.00)	0.0	48.0
Number of nocturnal traffic noise events	297.22 (139.32)	45	924	67.32 (20.43)	8	183	36.29 (16.69)	0	134
Annoyance due to traffic noise of the previous night (5-point scale)	1.76 (0.82)	1	5	1.64 (0.55)	1	4	1.46 (0.48)	1	5
General adaptation to chronic noise exposure of the respective traffic source (5-point scale)	3.21 (1.01)	1	5	3.03 (1.21)	1	5	2.58 (1.02)	1	5
Perceived noise load in the previous night (5-point scale)	2.07 (0.88)	1	5						
Subjective sleep quality in the previous night (0–60)	38.10 (7.18)	9.1	54.1						
Assessment of road transport (0–15)	8.9 (2.8)	3	13						

Study on road traffic, N = 40; study on railway traffic, N = 33; study on aircraft traffic, N = 64. Mean values, standard deviations (SD), and ranges of the predictor variables (Min./Max.) and the resulting annoyance responses.

**Table 2:** Random effects logistic regression models GEE 1 and GEE 2 calculated by Generalized Estimating Equation with regard to the effect of acoustical and non-acoustical predictors on annoyance.

		**Intercept**	***L_Aeq,night_* of Traffic Noise Events Indoors (dB)**	**Subjectively Perceived Noise Load in the Previous Night** **(5-Point Scale)**	**Subjective Sleep Quality in the Previous Night** **(0–60)**
GEE 1	Estimate	−8.974	0.142	3.859	−0.067
QIC = 101.348	SE	1.952	0.050	0.671	0.026
	*p*-value	<0.001	0.005	<0.001	0.009
	OR		1.152	47.437	0.936
	OR 95% CI (lower)		1.044	12.75	0.890
	OR 95% CI (upper)		1.271	176.531	0.983
		**Intercept**	**Number of Nocturnal Traffic Noise Events**	**Assessment of Road Transport** **(0–15)**	
GEE 2	Estimate	1.627	0.003	−0.294	
QIC = 177.370	SE	0.780	0.001	0.085	
	*p*-value	0.037	0.038	0.001	
	OR		1.003	0.745	
	OR 95% CI (lower)		1.000	0.630	
	OR 95% CI (upper)		1.006	0.881	

**Table 3:** Pooled random effects logistic regression calculated stepwise by Generalized Estimating Equation with regard to the effect of the *L_Aeq,night_* of road traffic, railway, and aircraft noise measured inside the participants’ bedroom on annoyance: simple model GEE 3, intermediate model GEE 4, and final model GEE 5.

	Statistic	Intercept	Traffic Noise Source			*L_Aeq,night_* of Traffic Noise Events Per Night Indoors (dB)	General Adaptation to Chronic Noise Exposure of The Respective Traffic Source (5-Point Scale)
			Road	Railway	Aircraft		
GEE 3	Estimate	−0.702	0.649	0.682	Reference group		
QIC = 1228.286	SE	0.161	0.287	0.304			
	*p*-value	<0.001	0.024	0.025			
	OR		1.914	1.978			
	OR 95% CI (lower)		1.089	1.091			
	OR 95% CI (upper)		3.362	3.586			
GEE 4	Estimate	−1.861	0.566	0.193		0.045	
QIC = 1216.632	SE	0.382	0.291	0.334		0.012	
	*p*-value	<0.001	0.052	0.563		<0.001	
	OR		1.761	1.213		1.046	
	OR 95% CI (lower)		0.995	0.630		1.021	
	OR 95% CI (upper)		3.114	2.334		1.071	
GEE 5	Estimate	−0.240	1.098	0.591		0.043	−0.639
QIC = 1129.012	SE	0.473	0.328	0.338		0.012	0.128
	*p*-value	0.611	0.001	0.081		0.001	<0.001
	OR		2.998	1.805		1.044	0.528
	OR 95% CI (lower)		1.577	0.930		1.019	0.411
	OR 95% CI (upper)		5.700	3.503		1.069	0.678

**Table 4:** Pooled random effects logistic regression calculated stepwise by Generalized Estimating Equation with regard to the effect of the number of nocturnal road traffic, railway, and aircraft noise events on annoyance: intermediate model GEE 6 and final model GEE 7.

	Statistic	Intercept	Traffic Noise Source			Number of Nocturnal Traffic Noise Events Per Night	Road x Number of Nocturnal Traffic Noise Events Per Night	Railway x Number of Nocturnal Traffic Noise Events Per Night	Aircraft x Number of Nocturnal Traffic Noise Events Per Night	General Adaptation to Chronic Noise Exposure of The Respective Traffic Source (5-Point Scale)
			Road	Railway	Aircraft					
GEE 6	Estimate	−1.363	0.715	0.845		0.017	−0.016	−0.010		
QIC = 1169.547	SE	0.249	0.537	0.553	Reference group	0.004	0.005	0.007	Reference group	
	*p*-value	<0.001	0.183	0.126		<0.001	0.001	0.153		
	OR		2.044	2.328		1.017	0.985	0.990		
	OR 95% CI (lower)		0.713	0.788		1.009	0.976	0.977		
	OR 95% CI (upper)		5.859	6.882		1.026	0.993	1.004		
GEE 7	Estimate	0.212	1.327	1.256		0.019	−0.017	−0.011		−0.665
QIC = 1080.203	SE	0.371	0.614	0.585		0.004	0.005	0.007		0.131
	*p*-value	0.567	0.031	0.032		<0.001	<0.001	0.152		<0.001
	OR		3.770	3.510		1.019	0.983	0.989		0.514
	OR 95% CI (lower)		1.131	1.115		1.010	0.974	0.975		0.398
	OR 95% CI (upper)		12.563	11.004		1.028	0.992	1.004		0.664

## Data Availability

Not applicable.

## Supplementary Materials

The following are available online at https://www.mdpi.com/article/10.3390/ijerph18094647/s1, Table S1: Averaged road traffic noise exposure and road traffic composition over the study nights for the individual time in bed for every participant, Table S2: Acoustical variables of nocturnal road traffic noise exposure and their association with the binary annoyance variable. Results are generated by Generalized Estimating Equations (GEE) analyses, Table S3: Non-acoustical parameters and their association with the binary annoyance variable generated by GEE analyses, Table S4: Main characteristics of the current study and those referred to within the discussion and conclusions.
