# Supplementary material for: Short-Term Annoyance Due to Night-Time Road, Railway, and Air Traffic Noise: Role of the Noise Source, the Acoustical Metric, and Non-Acoustical Factors
Source: Int J Environ Res Public Health. 2021 Apr 27;18(9):4647. doi: 10.3390/ijerph18094647 (PMC8123875; doi:10.3390/ijerph18094647)
Supplement: Supplementary file 1 [file ijerph-18-04647-s001.zip › ijerph-1184470/Table S1.pdf]

Table S1. Averaged road traffic noise exposure and road traffic composition over the study nights for the individual time in bed for every participant.

ID	Window position	Mean (SD) of $L_{Aeq,night}$ of traffic noise indoors	Mean (SD) of number of road traffic events	Road traffic composition in %			
				Passenger car	Van	Truck/Bus	Two-wheeler
1	closed	19.58 (.19)	178.25 (21.64)	70.97	13.04	8.56	7.43
4	tilted	28.08 (.67)	171.75 (19.31)	64.19	18.63	15.14	2.04
22	tilted	29.83 (1.1)	122.75 (6.19)	74.75	8.76	9.37	7.13
27	alternating	27.05 (4.31)	128.75 (17.63)	32.04	37.09	29.71	1.17
42 + 43	tilted	31.73 (.43)	215.75 (42.60)	60.95	22.36	12.98	3.71
57 + 58	alternating	24.83 (7.0)	224.50 (37.01)	77.39	13.25	7.68	1.67
213	closed	25.65 (1.77)	296.25 (54.74)	64.47	13.67	20.34	1.52
249 + 250	tilted	25.93 (4.16)	130.67 (2.89)	61.74	8.93	16.84	12.5
263	closed	26.25 (.79)	308 (67.56)	72.48	13.34	10.8	4.38
273	tilted	32.55 (1.86)	434 (41.47)	53.69	15.96	28.34	2.02
274	tilted	30.1 (2.47)	458 (227.554)	69.43	22	5.73	2.84
276	alternating	31.07 (8.93)	275.33 (162.792)	38.02	23.36	37.41	1.21
291	tilted	28.75 (1.44)	No data available				
303	closed	23.4 (2.22)	214.5 (80.04)	62.94	15.5	17.72	3.85
304	closed	19.6 (1.13)	98.75 (66.01)	39.75	26.84	23.54	9.87
307 + 308	tilted	30.78 (1.67)	341 (6.27)	72.8	14.88	9.68	2.64
316	alternating	43.85 (.66)	412.25 (18.19)	71.01	11.89	12.98	4.12
318	tilted	35.23 (2.3)	365.75 (34.99)	65.41	22.62	6.63	5.33
324	tilted	29.75 (2.38)	471.75 (42.14)	58.13	18.92	5.14	4.13
340	closed	23.58 (1.4)	357.75 (60.13)	74.42	15.02	5.52	5.03
343	tilted	26.2 (1.41)	192.5 (55.98)	54.55	28.05	5.06	12.34
344	closed	21.18 (.72)	No data available				
351	tilted	24 (.61)	259 (34.70)	72.85	13.64	6.82	6.69
356	open	38.15 (.8)	290.5 (22.49)	60.24	20.57	13.51	5.68

Table S1. (continued)

ID	Window position	Mean (SD) of $L_{Aeq,night}$ of traffic noise indoors	Mean (SD) of number of road traffic events	Road traffic composition in %			
				Passenger car	Van	Truck/Bus	Two-wheeler
357	closed	21.33 (.51)	295.25 (29.47)	66.64	13.63	13.97	5.76
368	closed	23.93 (.83)	221.25 (36.77)	73.45	9.15	14.69	2.71
372	closed	21.78 (.69)	469 (71.73)	77.56	12.74	3.20	6.50
375	tilted	26.63 (1.21)	130.5 (7.68)	69.92	1.72	11.30	17.05
376	closed	25.7 (2.86)	444 (139.16)	72.97	13.63	6.02	7.38
377	closed	26.15 (.93)	554.75 (59.03)	62.33	15.68	19.69	2.3
381	closed	27.08 (2.47)	700 (223.09)	61.43	17.43	18.32	2.82
384	tilted	34.23 (.99)	208 (68.40)	75.12	16.35	3.73	4.81
390	closed	27.68 (.63)	463.25 (80.47)	79.76	10.9	6.53	2.81
395	closed	23.35 (.68)	353.5 (33.81)	78.64	11.74	8.63	3.82
400	alternating	37.6 (.83)	No data available				
435	closed	25.33 (.92)	No data available				

$L_{Aeqnight}$ was derived from the acoustical recordings of 156 nights. Number of nocturnal road traffic events and its percentual composition was derived from the information of 139 nights provided by the traffic counter. Two ID-numbers in one cell mean that participants were investigated at the same location, same date and same time in bed. An alternating window position means that participants changed the position (open, closed, tilted) over the study nights. Two-wheeler contains motorized vehicles, but also bicicles. ID means the number of participant.
