# Supplementary material for: Short-Term Annoyance Due to Night-Time Road, Railway, and Air Traffic Noise: Role of the Noise Source, the Acoustical Metric, and Non-Acoustical Factors
Source: Int J Environ Res Public Health. 2021 Apr 27;18(9):4647. doi: 10.3390/ijerph18094647 (PMC8123875; doi:10.3390/ijerph18094647)
Supplement: Supplementary file 1 [file ijerph-18-04647-s001.zip › ijerph-1184470/Table S2.pdf]

Table S2. Acoustical variables of nocturnal road traffic noise exposure and their association with the binary annoyance variable. Results are generated by Generalized Estimating Equations (GEE) analyses.

Acoustical variable	Description	Estimate (SE)	p value	OR	OR 95% CI [lower, upper]	QIC
$L_{Aeq,night}$	A-weighted energy equivalent sound pressure level (L_{Aeq}) of road traffic noise exposure inside bedroom throughout time in bed in dB. This value was based on calculations of the acoustic data recorded inside*.	0.057 (0.027)	0.037	1.058	1.003, 1.116	216.265
Number of nocturnal road traffic noise events	Number of road traffic noise events based on information of traffic counter	0.002 (0.001)	0.118	1.002	1.000, 1.004	192.414
Total duration of nocturnal road traffic noise [min]	Overall duration of traffic noise exposure per night in minutes based on calculations of the acoustic data recorded inside*.	0.001 (0.006)	0.894	1.001	.989, 1.012	223.336
SNR_{night}	Signal to Noise Ratio across the night, with $L_{Aeq,night}$ defined as “signal” and the background noise of the whole night defined as “noise” in dB. This value was based on calculations of the acoustic data recorded inside*.	0.658 (1.271)	0.604	1.932	0.160, 23.317	222.734
$max\ L_{Amax,night}$	For each single pass-by per night, the maximum sound pressure level was computed (L_{Amax}). This value is the maximum of L_{Amax} of all pass-bys per night in dB based on calculations of the acoustic data recorded inside*.	0.014 (0.020)	0.441	1.014	0.978, 1.052	220.603
mean $L_{Amax,night}$	The mean of L_{Amax} of all pass-bys per night in dB based on calculations of the acoustic data recorded inside*.	0.039 (0.023)	0.082	1.040	0.995, 1.087	217.593
$NAT_{25,night}$	Number of pass-bys with a maximum level > 25 dB per night based on calculations of the acoustic data recorded inside*.	0.003 (0.002)	0.212	1.003	0.998, 1.007	220.902
$NAT_{30,night}$	Number of pass-bys with a maximum level > 30 dB per night based on calculations of the acoustic data recorded inside*.	0.003 (0.002)	0.125	1.003	0.999, 1.007	217.522
$NAT_{35,night}$	Number of pass-bys with a maximum level > 35 dB per night based on calculations of the acoustic data recorded inside*.	0.003 (0.002)	0.111	1.003	0.999, 1.006	215.771
$NAT_{40,night}$	Number of pass-bys with a maximum level > 40 dB per night based on calculations of the acoustic data recorded inside*.	0.002 (0.002)	0.193	1.002	0.999, 1.005	218.055
$NAT_{45,night}$	Number of pass-bys with a maximum level > 45 dB per night based on calculations of the acoustic data recorded inside*.	0.002 (0.002)	0.185	1.002	0.999, 1.005	219.187

Table S2. (continued)

Acoustical variable	Description	Estimate (SE)	p value	OR	OR 95% CI [lower, upper]	QIC
$NAT_{50,night}$	Number of pass-bys with a maximum level > 50 dB per night based on calculations of the acoustic data recorded inside*.	0.002 (0.002)	0.343	1.002	0.998, 1.005	219.978
$NAT_{55,night}$	Number of pass-bys with a maximum level > 55 dB per night based on calculations of the recorded acoustic data*.	0.001 (0.002)	0.685	1.001	0.997, 1.004	220.579
$NAT_{60,night}$	Number of pass-bys with a maximum level > 60 dB per night based on calculations of the acoustic data recorded inside*.	0.020 (0.011)	0.061	1.021	0.999, 1.043	219.772
$NAT_{65,night}$	Number of pass-bys with a maximum level > 65 dB per night based on calculations of the acoustic data recorded inside*.	0.135 (0.080)	0.093	1.144	0.978, 1.339	220.142

* Contrary to the traffic counter, the acoustic program was not able to evaluate several closely spaced road traffic events. Therefore, those events were classified as vehicle columns and counted as one noise event.
