# Supplementary material for: Short-Term Annoyance Due to Night-Time Road, Railway, and Air Traffic Noise: Role of the Noise Source, the Acoustical Metric, and Non-Acoustical Factors
Source: Int J Environ Res Public Health. 2021 Apr 27;18(9):4647. doi: 10.3390/ijerph18094647 (PMC8123875; doi:10.3390/ijerph18094647)
Supplement: Supplementary file 1 [file ijerph-18-04647-s001.zip › ijerph-1184470/Table S3.pdf]

**Table S3.** Non-acoustical parameters and their association with the binary annoyance variable generated by GEE analyses.

| Non-acoustical parameters | Description | Estimate (SE) | p value | OR | OR 95% CI [lower, upper] | QIC |
|---|---|---|---|---|---|---|
| Residential satisfaction [13-65] | Thirteen items that asked for satisfaction with the following aspects of the residential area ("1 = not at all" to "5 = extremely"): the residential area in general, the visual appearance, the neighbors, the air quality, the connection to public transport, proximity to the workplace, the quiet of the residential area, the shopping opportunities, the recreation and relaxation opportunities, and the loudness of road traffic, railway traffic, air traffic, and neighbors | -0.061 (0.029) | 0.037 | 0.940 | 0.888, 0.996 | 215.900 |
| General perception of loudness in the residential area [1-4] | One item ("1 = very loud" to "not loud at all"): "How loud do you perceive the residential area you live in?" | 1.434 (0.370) | <0.001 | 4.197 | 2.032, 8.669 | 203.063 |
| Subjective sleep quality [0-60] | Six 100 mm long visual analogue scales asking for difficulties in falling asleep (easy – difficult), calmness of sleep (calm – restless), sleep duration (too short – too long), restoration (high – low), sleep depth (sound – shallow), and body movements (little – much) [25] | -0.117 (0.024) | <0.001 | 0.889 | 0.848, 0.932 | 194.367 |
| General adaptation to chronic road traffic noise exposure noise [1-5] | One item ("1 = not at all" to "5 = extremely"): "How well can you adapt to road traffic noise in general?" | -0.596 (0.233) | 0.011 | 0.551 | 0.349, 0.870 | 211.964 |
| Assessment of road transport [3-15] | Three items ("1 = not at all" to "5 = extremely"): "Do you consider road transport as…" "…necessary?", "…acceptable?", "…economically important?" | -0.261 (0.077) | 0.001 | 0.770 | 0.662, 0.896 | 206.914 |
| Concerns about harmful effects of road transport [6-30] | Six items ("1 = not at all" to "5 = extremely"): "Do you consider road transport as…" "…dangerous for residents?", "…harmful to health of residents?", "…harmful to air quality?", "…harmful to the climate?", "…harmful to your own health?", and the feeling of threat by a nearby accident | 0.098 (0.047) | 0.036 | 1.103 | 1.006, 1.209 | 215.927 |
| Subjectively perceived noise load in the previous night [1-5] | One item ("1 = not at all" to "5 = extremely"): "Thinking about the last night, when you are at home, how intensely did you hear the road traffic noise?" | 3.381 (0.723) | <0.001 | 29.402 | 7.132, 121.210 | 112.834 |
| Long-term annoyance due to road traffic noise in general [1-5] | One item ("1 = not at all" to "5 = extremely"): "Thinking about the last 12 months, when you are at home, how much did noise from the following noise source bother, disturb, or annoy you?" "…road transport in general" [24] | 0.917 (0.320) | 0.004 | 2.502 | 1.337, 4.682 | 207.544 |

**Table S3.** (continued)

| Non-acoustical parameters | Description | Estimate (SE) | p value | OR | OR 95% CI [lower, upper] | QIC |
|---|---|---|---|---|---|---|
| Long-term annoyance due to noise by passenger car transport [1-5] | One item ("1 = not at all" to "5 = extremely"): "Thinking about the last 12 months, when you are at home, how much did noise from the following noise source bother, disturb, or annoy you?" "…passenger car transport" [24] | 0.610 (0.273) | 0.025 | 1.840 | 1.078, 3.140 | 214.713 |
| Long-term annoyance due to noise by heavy vehicles [1-5] | One item ("1 = not at all" to "5 = extremely"): "Thinking about the last 12 months, when you are at home, how much did noise from the following noise source bother, disturb, or annoy you?" "…heavy vehicles (truck, transporter, etc.)" [24] | 0.636 (0.198) | 0.001 | 1.890 | 1.283, 2.783 | 208.105 |
| Activity disturbances due to road traffic noise [12-60] | Twelve items referring to the past 12 months that asked for disturbance and annoyance due to road traffic noise during the following situations inside and outside, respectively ("1 = not at all" to "5 = extremely"): communication, mental activity, physical activity, relaxation, social meeting as well as for dinner inside and watching TV inside | 0.124 (0.041) | 0.002 | 1.132 | 1.046, 1.226 | 206.138 |
| Sleep disturbances [3-15] | Three items referring to the past 12 months that asked for disturbance and annoyance due to road traffic noise during the following situations ("1 = not at all" to "5 = extremely"): falling asleep, sleep in the first half of the night, and sleep in the second half of the night | 0.469 (0.115) | <0.001 | 1.598 | 1.274, 2.004 | 192.845 |
