# Supplementary material for: Short-Term Annoyance Due to Night-Time Road, Railway, and Air Traffic Noise: Role of the Noise Source, the Acoustical Metric, and Non-Acoustical Factors
Source: Int J Environ Res Public Health. 2021 Apr 27;18(9):4647. doi: 10.3390/ijerph18094647 (PMC8123875; doi:10.3390/ijerph18094647)
Supplement: Supplementary file 1 [file ijerph-18-04647-s001.zip › ijerph-1184470/Table S4.pdf]

Table S4. Main characteristics of the current study and those referred to within the discussion and conclusions.

Publication	Study setting	Traffic noise source	Acoustical variables	Noise ranges	Non-acoustical variables	Outcome
Current study	Field study	Road, rail, aircraft	$L_{Aeq,night}$, number of events, etc.	$L_{Aeq,\mathrm{night}}$: 19 – 49 dB, number of events: 0 - 924	Sleep quality, adaptation to traffic noise, assessment of transportation source, etc.	Short-term annoyance
[8]	Meta-Analysis	Aircraft, road, railway	Day–night level (*DNL*), day–evening–night level (*DENL*) calculated for outside	*DENL* and *DNL*: 45 – 75 dB(A)	-	Long-term annoyance
[11]	Questionnaire survey	Road, railway, aircraft	*DENL*, Intermittency Ratio (*IR*) calculated for outside	*DENL*: 30 – 85 B(A)	Age, sex, education level, etc.	Long-term annoyance
[12]	Field study	Railway, aircraft	Number of events, duration of events, L_{Aeq}, etc. measured inside	Number of railway events: 0 - 150	Age, gender	Short-term annoyance
[15]	Questionnaire survey	Road	*DENL*, number of events, maximal noise level, $L_{eq,night}$, etc. measured outside	$L_{Aeq,night}$: 32 – 74 dB	Subjective noise sensitivity, age, length of residence, etc.	Long-term annoyance
[16]	Field study	Railway	$L_{Aeq,night}$, number of events measured inside	Number of events: 8 - 183	Adaptation to traffic noise, subjective perception of noise load, length of residence, noise sensitivity, etc.	Short-term annoyance, self-reported sleep disturbances, pre-annoyance
[17]	Laboratory and field study	Aircraft	$L_{Aeq,night}$, number of events measured inside	$L_{Aeq,night}$: approximately 20 – 50 dB, number of events: 4 - 100	Age, adaption to traffic noise, pre-annoyance due to traffic noise, etc.	Short-term annoyance

Table S4. (continued)

Publication	Study setting	Traffic noise source	Acoustical variables	Noise ranges	Non-acoustical variables	Outcome
[19]	Field study	Aircraft	$L_{Aeq,night}$, number of events	$L_{Aeq,night}$: 0 – 50 dB	Long-term annoyance, noise sensitivity, adaption to traffic noise, etc.	Short-term annoyance
[21]	Review	Aircraft	Maximum sound pressure level (L_{Amax}), L_{Aeq}, Sound-exposure level (*SEL)*	L_{Aeq}: 35 – 75 dB, L_{Amax}: 30 – 110 dB, *SEL*: < 75 – 95+ dB	Short-term annoyance, sleep disturbance, tiredness, etc.	Long-term annoyance, health effects, etc.
[22]	Field study	Aircraft	L_{Amax}, number of events, L_{Aeq}, etc. measured outside	-	Long-term annoyance, noise sensitivity, time of day, domestic noise insulation, etc.	Short-term annoyance
[34]	Field study	Road	L_{Aeq} measured inside and outside	Outside: < 45 -65+ dB(A)	Window position, sound insulation, etc.	Difference between external and internal sound level
[35]	Field study	Road, aircraft	L_{Amax} measured inside and outside	Outside: approximately 35 - 90 dB(A), inside: approximately 20 – 70 dB(A)	Window position	Difference between external and internal sound pressure level
[37]	Meta-Analysis	Aircraft	Number of movements derived from survey reports and airport data	-	-	Community Tolerance Level (*CTL*)
[38]	Review	Aircraft	Number of movements, *DNL*	-	-	*CTL*

Table S4. (continued)

Publication	Study setting	Traffic noise source	Acoustical variables	Noise ranges	Non-acoustical variables	Outcome
[39]	Laboratory study	Road	L_{Aeq}	44 - 64 dB(A)	Gender	Subjective and objective sleep quality, performance,
[40]	Field study	Road	Number of events above threshold, L_{Aeq} Measured outside	Number of events above threshold: 75 – 85 dB(A)	Noise sensitivity, medical symptoms, mood, etc.	Self-reported Sleep disturbance, Long-term annoyance
[41]	Laboratory study	Road	Number of events, Maximum L_{Aeq}	Number of events: 4 – 128, Maximum L_{Aeq}: 45 – 60 dB	Noise sensitivity	Mood, performance, self-reported sleep disturbance, etc.
[42]	Questionnaire survey	Railway	*DENL* calculated for outside	40 – 90 dB(A)	Assessment of transportation mode, concern about harmful effects, noise sensitivity, etc.	Long-term annoyance
[43]	Questionnaire survey	Road, railway	L_{Aeq} calculated for outside	-	Subjective perception of noise load, noise disturbance, noise sensitivity, etc.	Long-term annoyance
[44]	Field study	Aircraft	L_{Aeq}, number of events above threshold, etc. calculated for outside	L_{Aeq} daytime: 41 – 62 dB, nighttime: 24 – 57 dB	Long-term annoyance, noise disturbance, aircraft-related fears, etc.	Short-term annoyance
[45]	Meta-Analysis	Road, railway, aircraft	*DNL* calculated for outside	45 – 75 dB(A)	-	Long-term annoyance
[46]	Laboratory study	Road, railway, aircraft	L_{Amax}, frequency, L_{Aeq}	L_{Aeq}: 40 – 85 dB, L_{Amax}: 42 – 95 dB	Noise sensitivity, age, gender	Short-term annoyance

Table S4. (continued)

Publication	Study setting	Traffic noise source	Acoustical variables	Noise ranges	Non-acoustical variables	Outcome
[47]	Laboratory study	Road, railway, aircraft	L_{Amax}, L_{Aeq}, duration, rise time	L_{Amax}: 70 and 80 dB(A), L_{Aeq}: 48 – 64 dB	Noise sensitivity, general annoyance	Short-term annoyance
[48]	Questionnaire study	Road, railway, aircraft	-	-	Social status, owner vs tenant of the residence, martialmarital status	Long-term annoyance
[49]	Laboratory study	Road, railway	L_{Aeq}	50 – 55 dB(A)	-	Loudness
[50]	Meta-Analysis	Road, railway, aircraft	Noise and Number Index (NNI), L_{Aeq} calculated for outside	*NNI*: 15 – 65 dB, L_{Aeq}: 30 – 80 dB	Attitudes, timing of noise events, experience with noise source, etc.	Long-term annoyance
[51]	Laboratory study	Road	*SEL*, frequency,	Civil vehicles *SEL* indoors: 40 – 67 dB(A), *SEL* outdoors: 54 – 81 dB(A)	-	Short-term annoyance
[52]	Review	-	L_{Aeq}	-	Noise sensitivity, general attitudes, local circumstances, etc.	Short-term and long-term annoyance
